# Supplementary figures and images for: High DDT resistance without apparent association to kdr and Glutathione-S-transferase (GST) gene mutations in Aedes aegypti population at hotel compounds in Zanzibar
Source: PLoS Negl Trop Dis. 2022 May 16;16(5):e0010355. doi: 10.1371/journal.pntd.0010355 (PMC9109918; doi:10.1371/journal.pntd.0010355)

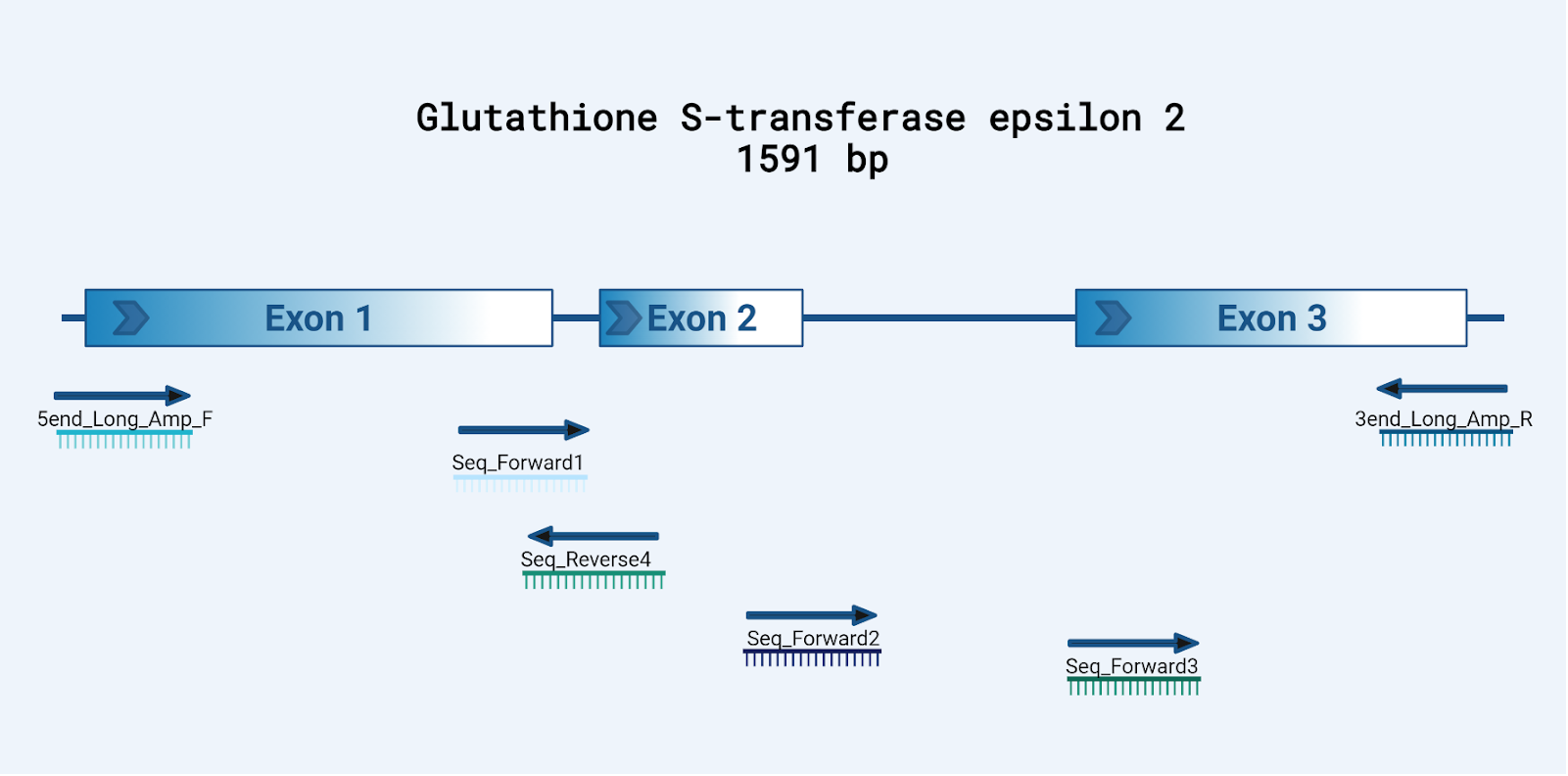

Supplement: S1 Fig — Arrows indicate direction of synthesis. (TIF) [file pntd.0010355.s001.tif]

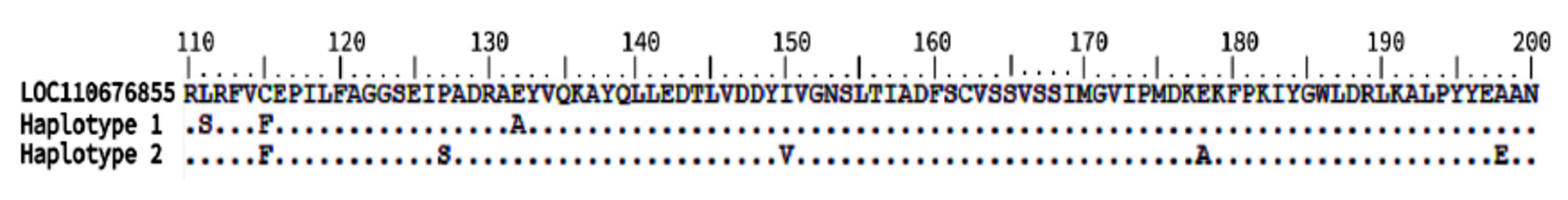

Supplement: S2 Fig — (TIFF) [file pntd.0010355.s002.tiff]
