# Supplementary material for: High DDT resistance without apparent association to kdr and Glutathione-S-transferase (GST) gene mutations in Aedes aegypti population at hotel compounds in Zanzibar
Source: PLoS Negl Trop Dis. 2022 May 16;16(5):e0010355. doi: 10.1371/journal.pntd.0010355 (PMC9109918; doi:10.1371/journal.pntd.0010355)
Supplement: S1 Table — (DOCX) [file pntd.0010355.s003.docx]

**S1_Table.** Mosquito control practices, type of insecticide, quantity and frequency of utilization at hotel compounds

| **Hotel** | **Anti-mosquito action** | **Site** | **Type of insecticides** | **Classe** | **Frequency of application** | **Quantity** |
| --- | --- | --- | --- | --- | --- | --- |
| Hotel A | Space spraying | Indoor | Propoxur/D-trans-allethrin  (250 mg/L) | Carbamate/Pyrethroid | Daily | 50 tin of 625 ml/month |
|  | Fumigation/Fogging | Indoor | Dichlorovos (500mg/L EC) | Organophosphate | Monthly | 400 litres/month |
|  | Fumigation/Fogging | Outdoor | Dimethoate (400mg/L EC) | Organophosphate | Monthly | 100 litres/month |
| Hotel B | Space spraying | Indoor | Propoxur/D-trans-allethrin  (250 mg/L) | Carbamate/Pyrethroid | Daily | 20 tin of 625 ml/month |
|  | Fumigation/Fogging | Indoor | Dichlorovos (500mg/L EC) | Organophosphate | Monthly | 20 litres/month |
|  | Fumigation/Fogging | outdoor | Deltamethrin (250 mg/L EC) | Pyrethroid | Fortynight | 20 litres/month |
| Hotel C | Space spraying | Indoor | Propoxur/D-trans-allethrin (250 mg/L) | Carbamate/Pyrethroid | Daily | 20 tin of 625 ml/month |
| Hotel D | Space spraying | Indoor | Repellent | Unknown | Daily | 30 tin of 250 ml/month |
|  | Reppelent | Outdoor | Propoxur/D-trans-allethrin (250 mg/L) | Carbamate/Pyrethroid | Monthly | 10 bottles 625 ml/month |
| *Undisclosed chemical content | | |  |  |  |  |
