# Supplementary material for: High DDT resistance without apparent association to kdr and Glutathione-S-transferase (GST) gene mutations in Aedes aegypti population at hotel compounds in Zanzibar
Source: PLoS Negl Trop Dis. 2022 May 16;16(5):e0010355. doi: 10.1371/journal.pntd.0010355 (PMC9109918; doi:10.1371/journal.pntd.0010355)
Supplement: S2 Table — Primers used for allele-specific qPCR of the VGSC (A) and CNV assay of the GSTe2 gene (B). (DOCX) [file pntd.0010355.s004.docx]

**S2_Table**: Primers used for allele-specific qPCR of the VGSC (A) and CNV assay of the GSTe2 gene (B)

| **A)** | | | |
| --- | --- | --- | --- |
| **Primer** | **Sequence (5’- 3’)** | **Product Size** | **Ref.** |
| F1534 (for WT) | #TCTACTTTGTGTTCTTCATCATATT | 90 bp | [1] |
| 1534 (common rev) | TCTGCTCGTTGAAGTTGTCGAT |  |  |
| 1534C (for MT) | ##TCTACTTTGTGTTCTTCATCATGTG | 110 bp | [1] |
| 1534 (common rev) | TCTGCTCGTTGAAGTTGTCGAT |  |  |
| V1016 (for WT) | ##ACAAATTGTTTCCCACCCGCACCGG | 102 bp | [2] |
| 1016 (common rev) | GGATGAACCGAAATTGGACAAAAGC |  |  |
| 1016I (for MT) | #ACAAATTGTTTCCCACCCGCACTG | 82 bp | [3] |
| 1016 (common rev) | GGATGAACCGAAATTGGACAAAAGC |  |  |
| V1016 (rev WT) | ##CTCTACTTTGTGTTCTTCATCATGTG | 80 bp | [4] |
| 1016 (common for) | TCTGCTCGTTGAAGTTGTCGAT |  |  |
| 1016G (rev MT) | #CAGCAAGGCTAAGAAAAGGTTAACTC | 60 bp | [4] |
| 1016 (common for) | TCTGCTCGTTGAAGTTGTCGAT |  |  |
| V410 (for WT) | ##ATCTTCTTGGGTTCGTTCTACCGTG | 180 bp | [5] |
| 410 (common rev) | TTCTTCCTCGGCGGCCTCTT |  |  |
| 410L (for MT) | #ATCTTCTTGGGTTCGTTCTACCATT | 160 bp | [5] |
| 410 (common rev) | TTCTTCCTCGGCGGCCTCTT |  |  |
| Long 5’- tail | GCGGGCAGGGCGGCGGGGGCGGGGCC | 25 bp | NA |
| Short 5’- tail | GCGGGC | 5 bp | NA |
|  | | | |
| **B)** | | | |
| GSTE2CNV (for) | GCGGATTTCAGTTGCGTTTCG | 149 bp | This study |
| GSTE2CNV (rev) | ACCTGCTCGGCTCCACTTC |  |  |

#: short 5’- tail attached, ##: long 5’- tail attached

**References**

1. Linss JGB, Brito LP, Garcia GA, Araki AS, Bruno RV, Lima JBP, et al. Distribution and dissemination of the Val1016Ile and Phe1534Cys Kdr mutations in Aedes aegypti Brazilian natural populations. Parasites & vectors. 2014;7 1:25; doi: 10.1186/1756-3305-7-25. <https://doi.org/10.1186/1756-3305-7-25>.

2. Saavedra-Rodriguez K, Maloof FV, Campbell CL, Garcia-Rejon J, Lenhart A, Penilla P, et al. Parallel evolution of vgsc mutations at domains IS6, IIS6 and IIIS6 in pyrethroid resistant Aedes aegypti from Mexico. Scientific reports. 2018;8 1:6747; doi: 10.1038/s41598-018-25222-0. <https://www.ncbi.nlm.nih.gov/pubmed/29712956>.

3. Saavedra-Rodriguez K, Urdaneta-Marquez L, Rajatileka S, Moulton M, Flores AE, Fernandez-Salas I, et al. A mutation in the voltage-gated sodium channel gene associated with pyrethroid resistance in Latin American Aedes aegypti. Insect molecular biology. 2007;16 6:785-98; doi: 10.1111/j.1365-2583.2007.00774.x. <http://www.ncbi.nlm.nih.gov/pubmed/18093007>.

4. Stenhouse SA, Plernsub S, Yanola J, Lumjuan N, Dantrakool A, Choochote W, et al. Detection of the V1016G mutation in the voltage-gated sodium channel gene of Aedes aegypti (Diptera: Culicidae) by allele-specific PCR assay, and its distribution and effect on deltamethrin resistance in Thailand. Parasites & vectors. 2013;6 1:253; doi: 10.1186/1756-3305-6-253. <https://doi.org/10.1186/1756-3305-6-253>.

5. Villanueva-Segura K, Ponce-Garcia G, Lopez-Monroy B, Mora-Jasso E, Perales L, Gonzalez-Santillan FJ, et al. Multiplex PCR for simultaneous genotyping of kdr mutations V410L, V1016I and F1534C in Aedes aegypti (L.). Parasites & vectors. 2020;13 1:325; doi: 10.1186/s13071-020-04193-0. <https://doi.org/10.1186/s13071-020-04193-0>.
