# Supplementary material for: High DDT resistance without apparent association to kdr and Glutathione-S-transferase (GST) gene mutations in Aedes aegypti population at hotel compounds in Zanzibar
Source: PLoS Negl Trop Dis. 2022 May 16;16(5):e0010355. doi: 10.1371/journal.pntd.0010355 (PMC9109918; doi:10.1371/journal.pntd.0010355)
Supplement: S3 Table — Primers used to amplify and sequence partial segment 6 in domain I-IV (A) and whole GSTe2 gene (B). (DOCX) [file pntd.0010355.s005.docx]

**S3_Table**: Primers used to amplify and sequence partial segment 6 in domain I-IV (A) and whole GSTe2 gene (B)

| **A)** | | | | |
| --- | --- | --- | --- | --- |
| **Primer** | **Sequence 5’-3’** | **Target region** | **Product size** | **Ref.** |
| **DomI_F*+** | GATAATCCAAATTACGGGTATAC | Dom I, Seg. 6, Exon 9 and 10 | ~500 bp | [1] |
| **DomI_R*+** | TTCTTCCTCGGCGGCCTCTT | Dom I, Seg. 6 | ~500 bp | [1] |
|  |  | Exon 9 and 10 |  |  |
| **AaSCF20*** | GACAATGTGGATCGCTTCCC | Dom II, Seg. 6, Exon 20 and 21 | ~630 bp | [2] |
| **AaSCR21*** | GCAATCTGGCTTGTT AACTTG | Dom II, Seg. 6, Exon 20 and 21 | ~630 bp | [2] |
| **AaSCF3+** | GTGGAACTTCACCGACTTCA | Dom II Seg. 6 | NA | [2] |
|  |  | Exon 20 and 21 |  |  |
| **AaSCR22+** | TTCACGAACTTGAGCGCGTTG | Dom II Seg. 6 | NA | [2] |
|  |  | Exon 20 and 21 |  |  |
| **AaSCF7*+** | GAGAACTCGCCGATGAACTT | Dom III, Seg. 6, Exon 30, 31 and 32 | ~748 bp | [2] |
| **AaSCR7*** | GACGACGAAATCGAACAGGT | Dom III, Seg. 6, Exon 30, 31 and 32 | ~748 bp | [2] |
| **AaSCR8+** | TAGCTTTCGCGGCTTCTTC | Dom III, Seg. 6, Exon 30, 31 and 32 | NA | [2] |
| **AlSCF6*** | TCGAGAAGTACTTCGTGTCG | Dom IV, P-region connecting seg. 5 and 6 | ~280 bp | [2] |
|  |  |  |  |  |
| **B)** | | | | |
| **GSTE2_Amp_Forward*** | CCCCGCTCGTACTGTCTAT | GSTe2 gene | ~1760 | This study |
| **GSTE2_Amp_Reverse*** | CCATGAAGCGATTCTCAATCCTA | GSTe2 gene | ~1760 | This study |
| **GSTE2_Seq1_Forward+** | CTCGTTTGCGGTTTGTGTGT | GSTe2 gene | NA | This study |
| **GSTE2_Seq2_Forward+** | GAGGGTCTGAGATTCCAGCG | GSTe2 gene | NA | This study |
| **GSTE2_Seq3_Foward+** | GCAAACGAACCGGTCACAAA | GSTe2 gene | NA | This study |

*: Amplifying primer, +: Sequencing primer

**References**

1. Saavedra-Rodriguez K, Maloof FV, Campbell CL, Garcia-Rejon J, Lenhart A, Penilla P, et al. Parallel evolution of vgsc mutations at domains IS6, IIS6 and IIIS6 in pyrethroid resistant Aedes aegypti from Mexico. Scientific reports. 2018;8 1:6747; doi: 10.1038/s41598-018-25222-0. <https://www.ncbi.nlm.nih.gov/pubmed/29712956>.

2. Chung HH, Cheng IC, Chen YC, Lin C, Tomita T, Teng HJ. Voltage-gated sodium channel intron polymorphism and four mutations comprise six haplotypes in an Aedes aegypti population in Taiwan. PLoS neglected tropical diseases. 2019;13 3:e0007291; doi: 10.1371/journal.pntd.0007291. <http://www.ncbi.nlm.nih.gov/pubmed/30925149>.
