# Supplementary material for: High DDT resistance without apparent association to kdr and Glutathione-S-transferase (GST) gene mutations in Aedes aegypti population at hotel compounds in Zanzibar
Source: PLoS Negl Trop Dis. 2022 May 16;16(5):e0010355. doi: 10.1371/journal.pntd.0010355 (PMC9109918; doi:10.1371/journal.pntd.0010355)
Supplement: Attachment — Submitted filename: Kampango et al 2022_Response to reviewers_Plos Neglect.docx [file pntd.0010355.s008.docx]

**Response to reviewers**

Reviewer #1:

It is a good manuscript, well written and presented data and technically well executed study. Reporting some interesting information about the lack of tight association of known DDT resistance markers with resistance phenotypes. Perhaps some basic synergism data, in case still possible to include, would have provided some more insights into the possible mechanisms (detoxification vs target site resistance)

Thank you for your suggestion. Unfortunately, we didn’t perform synergist assay for this study and the main reason is though synergist assays can sometimes provide usefull information on the probable metabolic mechanisms associated with resistance phenotype, they also suffer from specificity bias (difficult to say that a given enzyme inhibitor provides a comparable inhibition rate of all detox enzymes from a given family) and, therefore, leading to difficulties to interpret synergist bioassay data. In contrast, kdr mutation genotyping provides sufficient information to support the presence of other mechanisms.

**Reviewer #2**

**Title**

**High DDT resistance contrasts with low frequency of kdr mutations and no evidence of Glutathione-S-transferase (GST) gene mutations in Aedes aegypti populations at hotel compounds in Zanzibar**

The title is too long. I would suggest re-write it especially the part " no evidence of Glutathione-S-transferase (GST) gene mutations in Aedes aegypti to be more attractive to the reader

The title has been rewritten

**Abstract**

**Global efforts to control Aedes -transmitted pathogens still rely heavily on insecticides.**

It is better to add Aedes mosquitoes -transmitted

Done

**We investigated the profile of insecticide susceptibility of Aedes aegypti mosquitoes occurring at selected hotel compounds on Zanzibar Island.**

The Aedes aegypti should be in *italic* form

Done

**At least 100 adults Ae. aegypti females from larvae collected at four hotel compounds were exposed to discriminant concentrations of DDT, permethrin, deltamethrin, propoxur and bendiocarb to determine their susceptibility profile**

The Aedes aegypti should be written in *italic* form

I would prefer writing the percentage pre each insecticides such as 1% DDT, 1% permethrib.. and so on

Done

**Additionally, we explored the possible involvement of Glutathione-STransferase gene** **( GSTe2 ) mutations for the observed resistance profile**

Remove the space ( GSTe2 )

Done

**In vivo resistance bioassay indicated that Ae. aegypti at studied sites are were highly resistant to DDT, mortality rate ranged from 26.3% to 55.3% and, moderately resistant to deltamethrin with a, mortality rate between 79% to and 100%**

Are or were is a grammatical mistake

Grammar has been corrected

**In vivo resistance bioassay indicated that Ae. aegypti at studied sites are were highly resistant to DDT**

The Aedes aegypti should be written in *italic* form

Done

**Introduction**

**Notably, some hotels report spraying high quantities of chemical insecticides to reduce mosquito exposure (Kampango et al. 2019, unpublished data).**

I would recommend to specify the family of Insecticides used in hotels such as pyrethroids, organophosphates or ….etc

Done

**The higher and frequent use of insecticides at hotels as compared to surrounding communities may increase the resistance selection pressure on local vector populations with serious implication in case of mosquito-borne diseases outbreaks in the region.**

The author should add reference for this statement

This is our prediction based on widely stablished knowledge that intense use of insecticide imposes selection pressure on targeted population favouring the emergence of resistant strains

**Material and methods**

**Sample processing and identification**

**Collected mosquito larvae and pupae were maintained at insectary environmental conditions of 27 ± 2°C and 75 ± 10% relative humidity until they emerged as adults [40]**

The author should indicate the food name provided to the larvae in insectary and photoperiod

Done

**Three to five days old adult female Ae. aegypti were exposed to a diagnostic dose of DDT (4%), permethrin (0.75%), deltamethrin (0.05%), propoxur (0.1%) and bendiocarb (0.1%).**

The author should determine and write the class of insecticides before each item for example dose of the organochlorine DDT, pyrethroids permethrin ….etc

Done

**However, mosquitoes can retain resistance to DDT over long periods.**

The reference should be written after this sentence

This statement is supported by the reference that has been included just in the following sentence which reads “field studies in India on the malaria vectors *An. stephensi* and *An. culicifacies* showed that these species had retained resistance to DDT thirty years after the use of this insecticide was officially terminated [56]”. A clear connection between the two sentences has been added.

**Discussion**

***In vivo* susceptibility bioassays indicated that *Ae. aegypti* populations at four hotel sites**

**were highly resistant to DDT**

I would prefer that author to add the class of insecticides before the name such as the Organochlorine DDT, moderately resistant to the pyrethroids deltamethrin…etc.

The insecticides classes have been already indicated in the methods section, as suggest. As such, we think it would be redundant repeating the same sentence here.

**However, mosquitoes can retain resistance to DDT over long periods.**

The author needs to add reference

Done

**Reference**

Please revise all references and make sure to use one format

Done

**Table 1**

I suggest adding replicate tube number next to total tested

For example, total tested /Replicate No.

100/4 this is because there’s some tests using more 100 tests

Done
